# AN EXPERIENCE ON LIVING DONOR LIVER TRANSPLANTATION FOR COLORECTAL LIVER METASTASIS IN SOUTH AMERICA: A NEW ERA IN TRANSPLANT ONCOLOGY

**DOI:** 10.1590/0102-672020230046e1764

**Published:** 2023-09-18

**Authors:** Eduardo de Souza Martins Fernandes, Gabrielle Oliveira de Souza, Eduardo Pinho Braga, Rodrigo Lopes Leite Furtado, Raphael Rodrigues Corrêa, Camila Tobias Queiroz, Felipe Pedreira Tavares de Mello, Camila Liberato Girão, Pal-Dag Line, Orlando Jorge Martins Torres

**Affiliations:** 1 Universidade Federal do Rio de Janeiro, Hospital São Lucas, Department of Gastrointestinal Surgery, Liver Transplant Unit – Rio de Janeiro (RJ), Brazil; 2 Oslo University Hospital, Department of Oncology and Transplantation Medicine – Oslo, Norway; 3 Universidade Federal do Maranhão, Hospital Universitário Presidente Dutra, Department of Gastrointestinal Surgery-Hepatopancreatobiliary and Liver Transplant Unit – São Luís (MA), Brazil.

**Keywords:** Colorectal Neoplasms, Liver, Neoplasm Metastasis, Liver Transplantation, Living Donors, Surgical Oncology, Neoplasias Colorretais, Fígado, Metástase Neoplásica, Transplante de Fígado, Doadores Vivos, Oncologia Cirúrgica

## Abstract

**BACKGROUND:**

Complete surgical resection is the treatment of choice for patients with liver metastases, but in some patients, it is not possible to obtain a complete R0 resection. Moreover, the recurrence rate is up to 75% after three years. After the experience of the Oslo group with cadaveric liver transplant, some centers are starting their experience with liver transplant for colorectal liver metastasis.

**AIMS:**

To present our initial experience with living donor liver transplant for colorectal liver metastasis.

**METHODS:**

From 2019 to 2022, four liver transplants were performed in patients with colorectal liver metastases according to the Oslo criteria.

**RESULTS:**

Four patients underwent living donor liver transplants, male/female ratio was 3:1, mean age 52.5 (42–68 years). All patients were included in Oslo criteria for liver transplant. Two patients had already been submitted to liver resection. The decision for liver transplant occurred after discussion with a multidisciplinary team. Three patients recurred after the procedure and the patient number 3 died after chemotherapy.

**CONCLUSIONS:**

Living donor liver transplant is a viable treatment option for colorectal liver metastasis in Brazil, due to a shortage of donors.

## RESUMO

**RACIONAL:**

A ressecção cirúrgica completa é o tratamento de escolha para pacientes com metástases hepáticas, mas em alguns pacientes não é possível obter uma completa ressecção R0. Além disso, a taxa de recorrência é de até 75% após 3 anos. Após a experiência do grupo de Oslo com transplante hepático cadavérico, alguns centros estão iniciando sua experiência com transplante hepático para metástase hepática colorretal.

**OBJETIVOS:**

Apresentar a experiência inicial com transplante de fígado de doador vivo para metástase hepática colorretal.

**MÉTODOS:**

De 2019 a 2022, foram quatro transplantes hepáticos em pacientes com metástases hepáticas colorretais, de acordo com os critérios de Oslo.

**RESULTADOS:**

Quatro pacientes foram submetidos a transplante hepático de doador vivo, a relação homem/mulher de 3:1, a idade média foi de 52,5 (42-68 anos). Todos os pacientes foram incluídos nos critérios de Oslo para transplante de fígado. Dois pacientes já haviam sido submetidos à ressecção hepática. A decisão pelo transplante hepático ocorreu após discussão com equipe multidisciplinar. Três pacientes recidivaram após o procedimento e o paciente número 3 morreu após a quimioterapia.

**CONCLUSÕES:**

O transplante de fígado com doador vivo é uma opção viável de tratamento para metástase hepática colorretal no Brasil, devido à escassez de doadores.

## INTRODUCTION

Colorectal cancer is one of the most prevalent cancer types with a 10% incidence worldwide^1^. At diagnosis, about half of patients have metastases to the liver, the most frequent site. Long-term survival as high as 60% in five years has been achieved with different forms of liver resection and optimal medical treatment, particularly in those patients with a favorable clinical risk score such as described by Fong and colleagues^2,4,9^. Unfortunately, up to 75% of patients are initially diagnosed with unresectable lesions, and a significant proportion of those originally resected have recurrent liver disease after three years^3^. Liver transplantation (LT), in properly selected patients, has been increasingly adopted as a potential therapy in this scenario, with recently published impressive outcomes since the SEcondary CAncer studies SECA-I and SECA-II^5^.

The best selection criteria are still under evaluation. The Oslo and Fong clinical scores and the evaluation of 18F-FDG PET-CT metabolic tumor volume (MTV) may help in the decision to offer transplantation, although with relative limitations^7,9^. Organ scarcity in many countries limits the adoption of deceased donor liver transplantation (DDLT), despite the accumulating data that survival benefits are greater than standard-of-care chemotherapy or at least comparable to other conventional non oncological transplant indications^2^. In this setting, extended criteria donor organs (ECD) or living donor liver transplantation (LDLT) are the main strategies to surpass these challenges^5^.

Herein, we describe the experience of four LDLT cases of unresectable liver metastasis. As the first case series published in South America, we discuss its potential benefits and hope to contribute to the emerging field of transplant oncology.

## METHODS

We present our initial experience with patients with unresectable colorectal liver metastasis, treated with LDLT in a single center in Rio de Janeiro, between 2019 and 2022. All cases were confirmed liver-only metastases in patients with treated primary non-right-sided colon cancer, wild-type Kirsten rat sarcoma viral oncogene (KRAS), Eastern Cooperative Oncology Group (ECOG) 0–1 performance status, and favorable response to chemotherapy.

Exploratory laparotomy was done on the recipients and no extrahepatic metastatic disease was confirmed. All of them received a right liver graft. The middle hepatic vein was preserved for the donor and a reconstruction of the V5 and V8 veins was performed with biological prosthesis from a cadaveric donor or artificial prosthesis. The patients signed the informed consent for this publication.

## RESULTS

All our patients were young and with good performance status. However, LT was proposed as a last-resort therapy. Two patients had already been submitted to hepatic resection. Oslo and Fong scores^9^ were higher than in other reports, but the decision to proceed with transplantation was shared between the clinical and surgical teams and the patients (Table 1). All four patients had synchronous liver metastasis at diagnosis. Postoperative complications were high, with Clavien-Dindo grades IIIb (50%) and V (25%). All patients were discharged from the hospital. Three required new surgical intervention and one died after a chemotherapy session. Three of them developed localized metastatic disease, one to the adrenals, one to the lungs and one to the liver, with no impact on survival so far, which correlates to what is commonly described. Clinical and oncological characteristics are described below (Table 2).

**Table 1:** Oslo and Fong scores^9^ of the patients.

Oslo score (0–4)	Fong clinical risk score (0–5)
Tumor diameter >5.5 cm	Largest tumor >5 cm
CEA >80 μg/L	CEA >200 μg/L
Less than two-year interval between primary resection and LT	Synchronous disease (primary to liver recurrence <12 months)
Progressive disease at time of LT	More than one liver metastasis

CEA: carcinoembryonic antigen; LT: liver transplantation.

**Table 2:** Clinical and oncological characteristics of the four patients.

Patient	1	2	3	4
Sex	M	M	F	M
Age	68	42	54	47
Synchronous liver metastasis	Yes	Yes	Yes	Yes
ECOG	1	0	1	1
Oslo score	3	3–4	4	3–4
Fong score	2	1–2	3	2
Largest tumor (cm)	3.2	3.8	5.5	5.1
CEA	8.3	48	26.5	181.7
MTV (cm3)	58	59	>500	41
KRAS	Wild	Wild	Wild	Wild
Chemotherapy/cycles	FOLFOX/12	FOLFOX-FOLFIRI/12		
Primary resection (month/year)	8/2017	6/2019	4/2021	6/2021
Liver transplant	04/2019	01/2021	04/2022	06/2022
Time interval (years)	2	1.5	<1	1
Positive lymph node	Yes	Yes	Yes	Yes
>1 lesion	Yes	Yes	Yes	Yes
Progressive disease	Yes	Yes	Yes	No
Recurrence	Yes	Yes	No	No
Site	Lung/Liver	Adrenal gland	-	-
Follow-up (years)	>3	2.5	1	<1
Outcome	Alive	Alive	Dead	Alive

ECOG: Eastern Cooperative Oncology Group score; CEA: carcinoembryonic antigen; MTV: metabolic tumor volume; KRAS: Kirsten rat sarcoma viral oncogene.

### Patient 1

**Figure 1:**
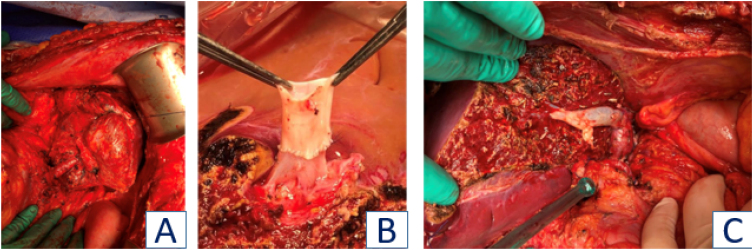
A. Recipient with multiple liver metastases; B. Reconstruction of middle hepatic vein with donor graft; C. Liver graft in the recipient.

**Figure 2:**
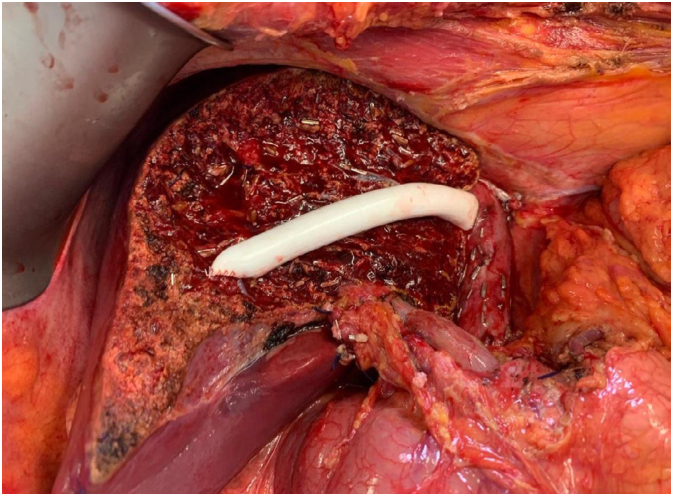
Prosthesis replacing the middle hepatic vein (Hong Kong technique^3^).

A 68-year-old male, ECOG 1, initially underwent a low anterior resection for a rectal tumor in November 2017. The diameter of the largest tumor was 3.1 cm. After 12 cycles of chemotherapy (FOLFOX), he was submitted to a left hepatectomy (lesions on segments IVa/V/VI) with radiofrequency ablation of two minor lesions on the right lobe in August 2018. Recurrence of several new lesions in the liver was diagnosed, deemed unresectable and LT was proposed. He underwent LDLT in April 2019. We used the right lobe without the middle hepatic vein (Figure 1). Reconstruction of the V5 and V8 veins was performed with artificial prosthesis and anastomosis in the vena cava with partial clamping of the vein by Hong Kong technique (Figure 2)^3^. The patient developed biliary leakage and needed drainage and a biliary prosthesis by interventional radiology. After four months he was diagnosed with biliary stricture and underwent a hepaticojejunostomy. In June 2020, a liver recurrence was diagnosed with a single lesion on segment VII. Radiofrequency ablation was performed. At present, he has liver, lung, and bone metastatic disease.

### Patient 2

The recipient was a 42-year-old male patient, ECOG 0, with multiple liver metastases of colorectal origin. The primary sigmoid colon tumor was diagnosed in July 2018 and the carcinoembryonic antigen (CEA) at the time was 43 ng/dL. He underwent LDLT in June 2019 and had no postoperative complications, being discharged after ten days. The patient then received neoadjuvant chemotherapy with the FOLFOX and FOLFIRI regimens in 12 cycles from July 2019 to January 2020. Afterward, he underwent a partial colectomy associated with left-lateral sectionectomy in February 2020. In March 2020, he performed a PET scan that revealed multiple lesions restricted to the liver. The possibility of LT was discussed, but a new cycle of chemotherapy was chosen. He received a new chemotherapy treatment with FOLFIRI and AVASTIN, in seven more cycles, between April and October 2020. In the end, another PET scan demonstrated restrictive liver disease with a significant reduction in FDG 18 uptake. In December 2020, magnetic resonance imaging with hepatic-specific contrast showed multiple lesions (the largest with a diameter of 3.9 cm in segment VII). The CEA in this period was 2.4 ng/dL. The patient underwent an adrenalectomy due to metastatic disease in July 2022 and is currently in adjuvant chemotherapy.

### Patient 3

**Figure 3:**
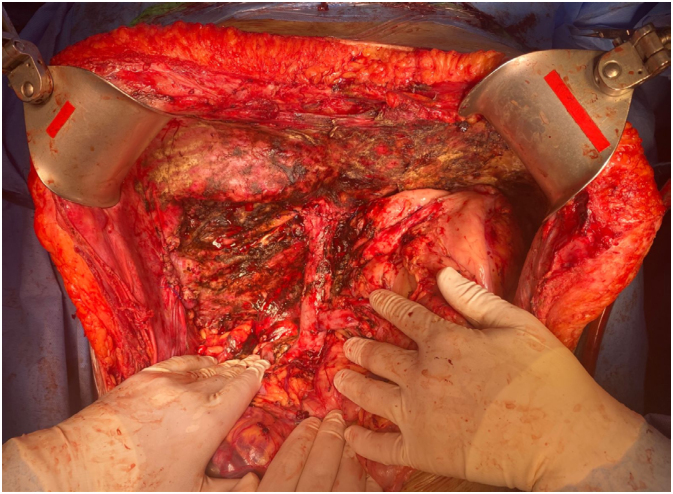
Patient 3 after total hepatectomy and portocaval anastomosis.

A 54-year-old female, ECOG 1, with multiple liver metastases of colorectal origin. She underwent a low anterior resection for sigmoid cancer in January 2021. We opted for systemic chemotherapy with FOLFOX and FOLFIRI (16 cycles) and posterior liver Transarterial Chemoembolization (TACE) in October 2021. In April 2022 she presented a large liver, high tumor burden, and CEA of 250 ng/dL. She presented with sudden and rapid deterioration of her clinical state with desaturation, lowered consciousness level, disorientation, and abdominal compartment syndrome. After stabilization, she went through an urgent exploratory laparotomy, and a total hepatectomy was performed with portocaval anastomosis (Figure 3). The total time of anhepatia before the LDLT was 12 hours. She had a satisfactory postoperative recovery, being discharged after 12 days. The patient was being treated with adjuvant chemotherapy but suddenly died a few days after a chemotherapy session in August 2022.

### Patient 4

A 47-year-old male, ECOG 1, diagnosed with colorectal tumor with synchronous liver metastasis. He underwent resection for the primary tumor in June 2021, and undertook chemotherapy with FOLFOX and AVASTIN (8 cycles) from July 2019 to December 2020, in addition to AVASTIN + XELODA (2 cycles). He presented decreasing levels of CEA and liver-only but unresectable metastatic disease. He underwent LDLT in June 2022 and developed biliary leakage with the need for drainage and a biliary prosthesis by interventional radiology 14 days after surgery. He required a hepaticojejunostomy due to biliary stricture that was performed during the same hospitalization. The patient had a good postoperative recovery and was discharged after 34 days of hospitalization. The patient has not reported any complications since then.

## DISCUSSION

LT as a treatment for hepatic metastasis of colorectal cancer started at the University of Vienna in the 1980s. It was discouraged after very unsatisfactory results, even though the significant cause of the high mortality rate was unrelated to the neoplastic disease^14^. More recently, the Oslo Group showed promising results through two studies. The SECA I, completed in 2013, included 21 highly selected patients based on liver tumor size greater than 5.5 cm, CEA level over 80 mg/dL, non-response to chemotherapy, and a short interval between TX and primary tumor resection. The patients’ ECOGs were 0–1, with restricted unresectable colorectal liver metastasis. The five-year survival of such patients was 60%^11,15^. The authors concluded that favorable outcomes are possible after LT for colorectal liver metastasis.

The most recent study, SECA II, included 15 patients who underwent an LDLT with stricter selection criteria, including SECA I plus PET-CT, used to calculate MTV for all liver metastases. Compared to SECA I, it was observed an increase of 23% in the five-year survival rate, suggesting that high metabolic activity and high tumor burden increase the possibility of micrometastases and extrahepatic metastases that may not have been previously detected^4,10,17,18^.

Despite all good results with LT to colorectal liver metastasis, the major challenge in establishing it as a formal treatment modality is the scarcity of available organs^6,8,19,20^. The use of extended criteria donor (ECD) submitted or not to liver machine perfusion and new surgery techniques such as the Resection And Partial Liver Transplantation With Delayed Total Hepatectomy) (RAPID) technique emerge as a way to reduce the disbalance between supply and demand^6,15,18^.

The latest effort to guide the indications of this oncological transplant modality was published in January 2021. The International Hepato-Pancreato-Biliary Association commissioned a multidisciplinary group of experts to develop a consensus and guidelines named Liver Transplantation for Colorectal Metastasis (LT-CoMet 21). The focus was to standardize the nomenclature and define the principles and management in the area, provided by an ethical scientific basis^12,13^.

In Brazil, according to legislation, LT from cadaveric donor for liver metastasis is prohibited, except for neuroendocrine tumors^16^. Conditioned by the risk to the donor, LDLT is the only modality to be offered to patients with liver colorectal metastasis. Therefore, it is essential to use clinical strict criteria to identify those patients with a chance of long overall survival after LT^2^. Colorectal liver metastasis should be acknowledged as a formal transplant indication.

## CONCLUSION

The rise of new surgical techniques and increased expertise in liver transplantation has expanded the applicability of cancer transplantation as a viable treatment option for colorectal liver metastasis. Unfortunately, our experience is still limited with a low number of patients and a short follow-up period, precluding statistical analysis and a more comprehensive investigation of data. This is still a novelty treatment in Brazil, and we believe transplantation is proposed much later than it should be in the treatment chain. More accurate patient selection can be achieved; the current clinical and imaging scores have several limitations, but they will come with the increasing experience of this treatment modality.
